# Polaron Diffusion in Pentathienoacene Crystals

**DOI:** 10.1038/s41598-020-63699-w

**Published:** 2020-05-06

**Authors:** Marcelo Lopes Pereira, Rafael Timóteo Sousa, William Ferreira Giozza, Luiz Antonio Ribeiro

**Affiliations:** 1 Institute of Physics, University of Brasília, Brasília, 70919-970 Brazil; 2 Department of Electrical Engineering, University of Brasília, Brasília, 70919-970 Brazil

**Keywords:** Materials science, Physics

## Abstract

Molecular crystals have been used as prototypes for studying the energetic and dynamic properties of charge carriers in organic electronics. The growing interest in oligoacenes and fused-ring oligothiophenes in the last two decades is due, in particular, to the success achieved in conceiving pentacene-based organic photovoltaic devices. In the present work, a one-dimensional Holstein-Peierls model is designed to study the temperature-dependent polaron transport in pentathienoacene (PTA) lattices. The tight-binding Hamiltonian employed here takes into account intra and intermolecular electron-lattice interactions. Results reveal that polarons in PTAs can be stable structures even at high temperatures, about 400 K. During the dynamical process, these charge carriers present a typical 1D random walk diffusive motion with a low activation energy of 13 meV and a room temperature diffusivity constant of 1.07 × 10^−3^ cm^2^ s^−1^. Importantly, these critical values for the polaron diffusion and activation energy are related to the choice of model parameters, which are adopted to describe pristine lattices.

## Introduction

Organic crystalline semiconductors have emerged in the last two decades as promising solutions in substituting Silicon and Gallium for the manufacture of electronic devices, especially those aimed to capture, emit, and control light^1–5^. Among the reasons for that stand out their lower environmental impact, good transparency, and flexibility^6,7^. Particularly, molecular crystals present two types of electron-phonon (e–ph) interactions, namely local intramolecular (Holstein-type) and non-local intermolecular (Peierls-type) interactions^8–10^. The Holstein-type^11,12^ is related to covalent bonds and modulates the site energy vibrations, and the Peierls-type, in turn, describes the modulation of the intermolecular vibrations governed by weak van der Waals interactions^13^. In these materials, polarons are the primary structures that play the role of the charge transporter^9,14^.

Recently, Zhang and colleagues have theoretically investigated the charge transport parameters and carrier mobilities in pentacene and pentathienoacene (PTA) crystals within the framework of Marcus’ semi-classical theory and quantum nuclear tunneling model, coupled with random walk simulation^15^. In their work, a systematic comparative study was also carried out for pentacene and PTA to gain insights into the theoretical design of these materials. The key finding in their results have revealed that pentacene and PTA present similar lattice structures, but they exhibit substantially different intrinsic transport properties. By using a similar approach, Takimiya *et al*. studied several high-mobility organic semiconductors (among them the PTA) to obtain their molecular factors and electronic structure, which would benefit the design strategies for the synthesis of molecules for new field-effect applications^16^. In an overall fashion, their results suggest that molecular design strategies should be based on the understanding of intermolecular orbital overlaps and their dimensionality in the crystal. Importantly, other relevant works have also used similar methods to study the diffusion of charge carriers and excitons in organic materials^17–24^. Albeit several works have used a quantum-mechanical description to describe the polaron dynamics^25–30^, a coherent quantum-mechanical description of polaron diffusion in PTA is still missing.

In the present work, we extend our very recent analysis of polaron properties in PTA^31^ by investigating its temperature-dependent dynamics in this class of material. The numerical approach employed here is based on a Holstein-Peierls Hamiltonian that takes into account both intra and intermolecular electron-lattice interactions to consider the presence of a polaron in a one-dimensional PTA lattice. We systematically investigate the impact of different thermal bath regimes on polaron stability and dynamics. In this sense, our results give information about diffusion parameters such as diffusion length, diffusivity, activation energy, as well as charge mobilities.

## Results

To characterize the polaron diffusion in PTA crystals, we adopt a one-dimensional lattice with 100 sites. The system dynamics take place during 5 ps for temperature regimes ranging from 50 to 400 K, with an increment of 50 K and 1000 realizations for each one of them. All the realizations start from the same initial state, a stable polaron on its ground state configuration^31^. Here, we use two measures to characterize the polaron stability: the polaron formation energy (*E*_*P*_) and the inverse participation ratio (IPR) related to the system’s charge density. *E*_*P*_ is the energy difference between the neutral ground state and relaxed confirmation (${E}^{\pm }$) energies in the presence of additional charge. In this sense, once the ground state and relaxed energies for a PTA lattice are obtained, the polaron formation energy is calculated as ${E}_{P}={J}_{0}-{E}^{\pm }$, where ${J}_{0}$ is the transfer integral between next-neighboring molecules in a pristine lattice^13^. For a lattice with ${J}_{0}\, > 0$, stable polarons take place when ${E}_{P}\, < 0$. We obtain ${E}_{P}=-\,39$ meV for a 1D PTA lattice, that agrees with values reported for other molecular crystals, that are about 100 meV or less^32,33^. It is worthwhile to stress that ${E}_{P} \sim {J}_{0}$ denotes a Fröhlich-like polaron solution^34^ (or large polaron)^34^, whereas ${E}_{p}\gg {J}_{0}$ stands for the Holstein-like polaron solution (or small polaron)^11^. As discussed in our previous researches, large polarons are dynamically stable and should be considered the primary quasiparticles when it comes to the charge transport mechanism in organic crystalline semiconductors^31,35–37^.

The IPR, in turn, is the quantity that measures how many sites share the additional charge, so that $0 < {\rm{IPR}} < 1$, and is given by

1 $${\rm{IPR}}=\frac{\sum _{j}{|{\rho }_{j}|}^{2}}{{(\sum _{j}{|{\rho }_{j}|}^{2})}^{2}},$$

where ${\rho }_{j}$ is the charge density of *j* site. The IPR related to a large polaron solution in a 2D PTA lattice may vary from 0.35 to 0.70 and the configuration of its ground state geometry was presented in our previous research^31^. For the 1D PTA lattice as modeled here, we obtain ${\rm{IPR}}=0.40$ that characterizes a stable large polaron.

**Figure 1:**
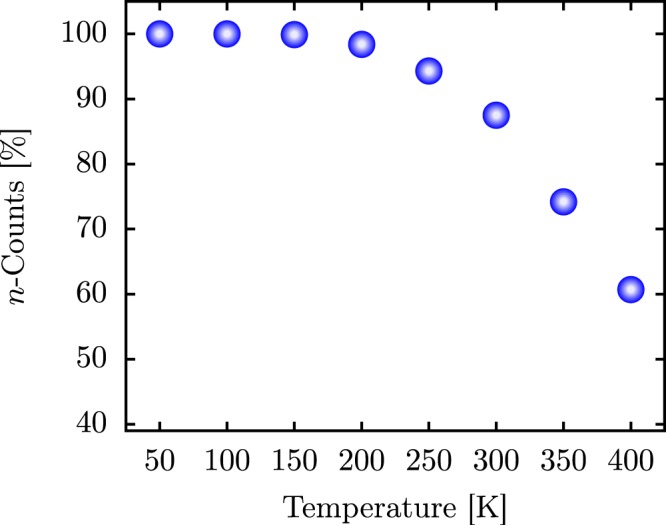
Temperature-dependent stability of large polarons in model 1D PTA lattices. The *n*-counts stands for the counting of stable polarons based on $\overline{{\rm{IPR}}}$ measurements.

Having presented the initial condition of the simulations, we now present the main features of the polaron diffusion in 1D PTA lattices. To do so, we begin by discussing the impact of the temperature on the polaron stability in these materials. Figure 1 depicts the counting of stable polarons (*n*-counts) for a given thermal bath having as reference the average of IPR ($\overline{{\rm{IPR}}}$). The $\overline{{\rm{IPR}}}$ is calculated using the IPR values obtained at each time step during the last 2 ps, neglecting, therefore, the initial polaron configuration that is the same for all realizations. As mentioned above, we count just stable large polarons, and they present IPR values ranging from 0.35 to 0.70. In this sense, Fig. 1 illustrates the percentage count of stable polarons over 1000 realizations in which $\overline{{\rm{IPR}}}$ lies in the range mentioned above for different thermal baths ranging in the interval 50–400 K with an increment of 50 K. From this figure, we can rapidly note that polarons tend to be less stable structures for temperatures higher than 150 K.

For thermal baths above this critical value, a considerable part of the energy related to the molecular vibrations, that are imposed by thermal random forces, is transferred to electrons. This mechanism of energy transfer between lattice and electrons takes place because of intra and intermolecular e-ph interactions. If this transfer continues unhindered, the charge decouples from the lattice and the composite state between charge and lattice deformations, which characterized the polaron, vanishes resulting in the dissociation of this quasiparticle.

For temperatures between 200 and 300 K, the polaron dissociates in few realizations so that more than 80% of them result in stable large polarons. For thermal baths between 300 and 400 K, more than 60% of realizations result in stable charge carriers, which suggests that polarons can be stable structures at room temperature regimes when it comes to 1D PTA lattices.

**Figure 2:**
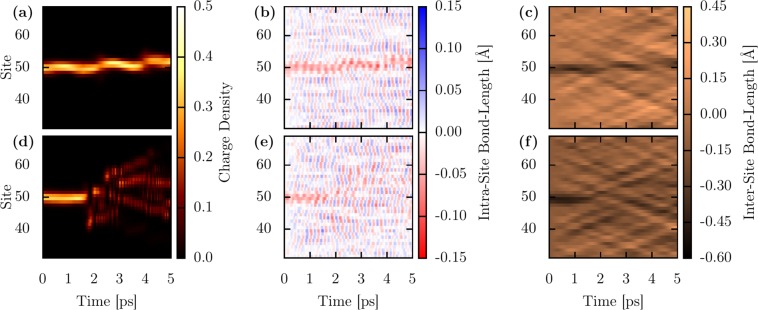
Polaron dynamics in a model 1D PTA lattice. Panels (a,d) depict the temporal evolution of the mean charge density, (**b,e**) and (**c,f**) show the temporal evolution for the intra (*u*_*j*_) and intermolecular (*v*_*j*_) displacements, respectively.

The trend for the temperature-dependent polaron motion in model 1D PTA lattices can be summarized in Fig. 2. Figure 2(a–f) illustrate the polaron dynamics for 150 K and 250 K, respectively. Particularly, Fig. 2(a,d) show the temporal evolution of the mean charge density, Fig. 2(b,e) and Fig. 2(c,f) depict the temporal evolution for the intra (*u*_*j*_) and intermolecular (*v*_*j*_) displacements, respectively. In Fig. 2(a) one can see that the molecular charge, initially centered at site 50, performs a random motion going back and forth among the lattice sites 50–53. The intra and intermolecular molecular vibrations impose such a random walk motion presented by the molecular charge. In Fig. 2(b), one can realize that there are localized red regions that follow the movement of the molecular charge. These regions denote local intramolecular compressions that are associated with the presence of the additional charge that forms the polaron. The apparent roughness in Fig. 2(b) illustrates how the intramolecular vibrations for the rest of the lattice behave during the time. Analogously, in Fig. 2(c), there are local black regions following the motion of the molecular charge. These distinct regions are local intermolecular compressions associated with the presence of a polaron in the lattice. It is clearly shown by Fig. 2(b,c) that the intra and intermolecular local deformations follow the motion of the molecular charge until the end of the simulations. This collective behavior between charge and lattice deformation presented during the system dynamics represents how the polaron can keep its integrity for small temperature regimes. A different case for the physical picture discussed above can be seen in Fig. 2(d–f), that shows the system dynamics for 250 K. In Fig. 2(d) it is possible to note that the initial sign for the charge localization that denotes the formation of a stable polaron breaks at 2 ps spreading charge through the lattice. From that moment, the charge decouples from the lattice and the local intra, and intermolecular compressions vanish, and a stable polaron is not present in this particular realization.

**Figure 3:**
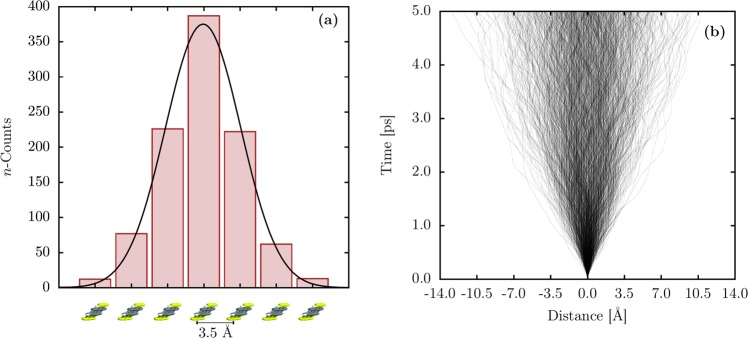
(**a**) Distribution of the of the polaron displacement regarding its initial position at 5 ps for 50 K. (**b**) Polaron trajectory for 1000 realizations.

The overall description of the polaron diffusion in PTA lattices can be achieved by studying an ensemble of its trajectories for a given thermal bath. In this sense, Fig. 3 illustrates the counting for the final position of the polaron (Fig. 3(a)) and its propagation (Fig. 3(b)) for 1000 realizations at 50 K. Figure 3(a) shows the polaron distribution regarding the distance from the origin (site 50) during 5 ps of simulation. Each bar Fig. 3(a) denotes the final position counting for the polaron, used to understand the critical limit for its diffusion in a PTA lattice. The bars are centered in molecules nearby the central unit in which the polaronic charge is placed, as represented in the bottom of Fig. 3. The Gaussian regression denotes that the average position for the polaron displacement is zero. This analysis can be used to derive the probability of finding a polaron at a region in the lattice for a certain thermal bath. The lines in Fig. 3(b) represent the polaron position as a function of time for a given realization. The polaron trajectory (*x*_*p*_(*t*)) is obtained by using the expression

2 $${x}_{p}(t)=\frac{n}{2\pi }{\rm{\arg }}\left(\mathop{\sum }\limits_{l=1}^{n}\,\exp \left(\frac{2\pi il}{n}\right)\times \bar{\rho }(t)\right)\times a,$$

where *n* is the total number of molecules, and *a* is the lattice constant^38^. Here, we set *a* = 3.5 Å. One can note that the polaron performs a typical Brownian motion. As mentioned above, the fluctuations in the polaron trajectory are imposed by the temperature effects. Moreover, one can see that there is a dispersion trend of the polaron path, suggesting a diffusive behavior.

**Figure 4:**
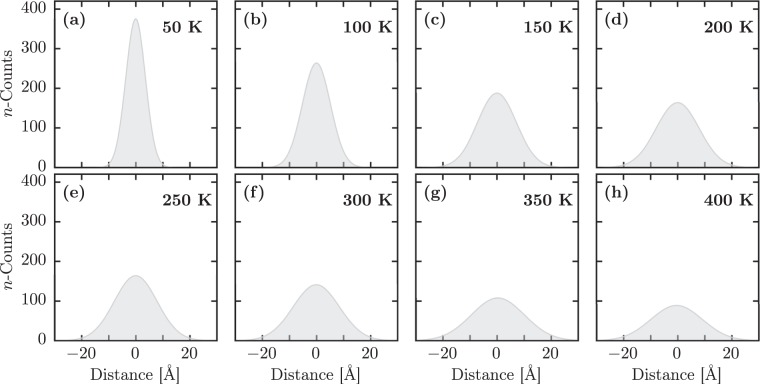
Distribution of the polaron displacement from its origin for the following thermal baths: (**a**) 50 K, (**b**) 100 K, (**c**) 150 K, (**d**) 200 K, (**e**) 250 K, (**f**) 300 K, (**g**) 350 K, and (**h**) 400 K.

Figure 4 shows how the temperature regimes used here impact the polaron diffusion in model 1D PTA lattices. In this figure, we can note that for higher temperatures, the polaron displacement from its origin increases. Accordingly, the counting for the zero displacement decreases proportionally. These results suggest that the temperature-dependent polaron transport in PTA lattices is limited to around twelve molecules. Importantly, the model system used here is a pristine lattice. Therefore, this critical limit for the polaron diffusion in real systems should be, in fact, substantially smaller due to, for instance, lattice defects and charge recombination that can reduce the mean free path of charge carriers in organic semiconductors^39,40^.

**Figure 5:**
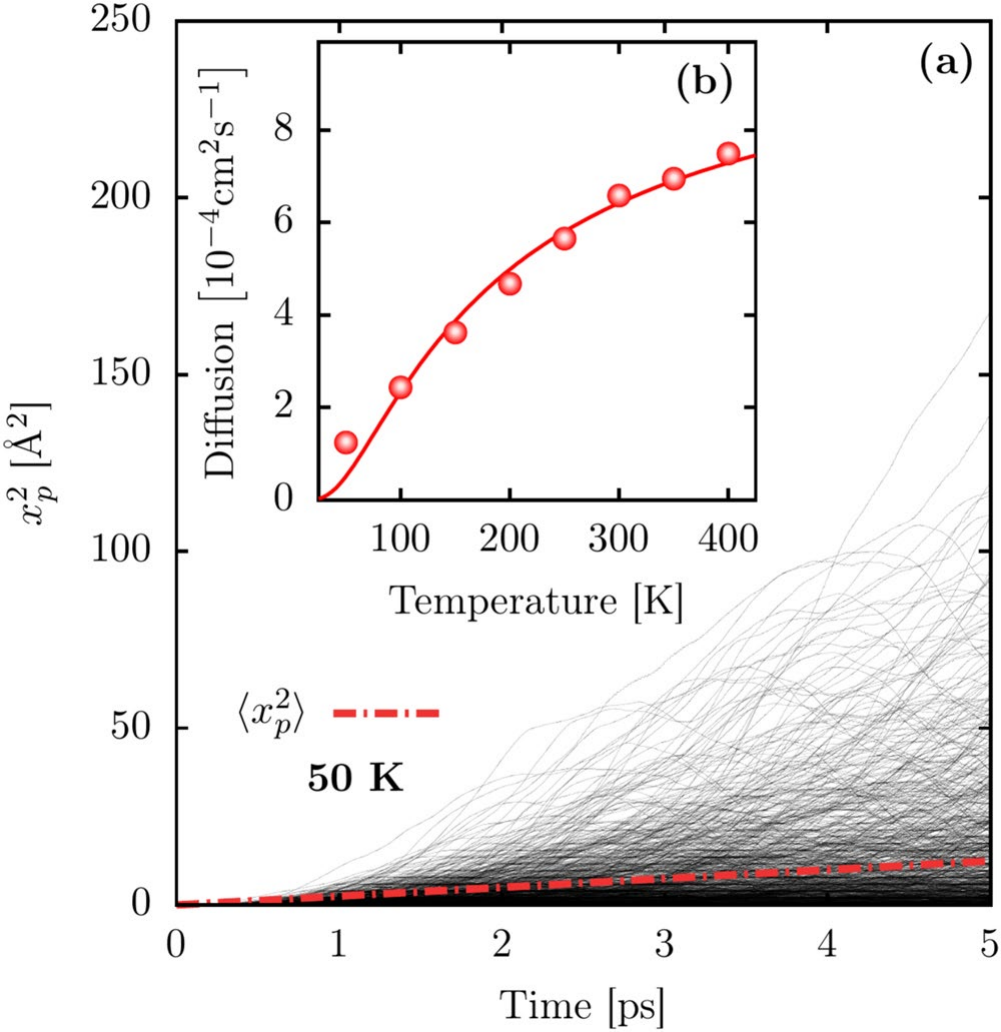
(**a**) Time-dependent squared displacement for the polaron transport in a 1D PTA lattice. (**b**) Temperature-dependent polaron diffusivity.

Now we can turn to the discussions about the polaron mobility in PTA lattices subjected to a thermal bath. To do so, Fig. 5(a)depicts the time evolution of the mean-square displacement for the polaron trajectories (${x}_{p}^{2}(t)$) presented in Fig. 3(b). The inset panel, Fig. 5(b), shows the regression for the temperature-dependent polaron diffusivity. The red dashed line in Fig. 5(a) establishes the linear regression over 1000 realizations. The angular coefficient of this regression stands the polaron diffusion according to the following expression

3 $$D=\frac{1}{2N}\mathop{\mathrm{lim}}\limits_{t\to \infty }\frac{\langle {x}_{p}^{2}(t)\rangle }{t},$$

where *N* is the system’s dimensionality. In addition, the obtained diffusion values are used to estimate the polaron mobility, *μ*, by using the Einstein relationship

4 $$\mu =\frac{e}{{k}_{B}T}D,$$

where $e$ is the electronic charge, ${k}_{B}$ is the Boltzmann’s constant, and *T* the temperature. The temperature-dependent polaron transport in organic crystalline semiconductors follows an Arrhenius type law

5 $$D(T)={D}_{0}\exp (\,-\,{E}_{A}/{k}_{B}T),$$

where ${D}_{0}$ is the maximum diffusion coefficient, and ${E}_{A}$ is the activation energy for the diffusion. As represented in Fig. 5(b), we have performed a regression to derive the polaron diffusivity. The values obtained are ${D}_{0}=1.07\times {10}^{-3}\,{{\rm{cm}}}^{2}{{\rm{s}}}^{-1}$ and ${E}_{A}=13.08$ meV. Furthermore, one can note in Fig. 5(b) that the diffusion ($D$) increases for higher temperature values. Importantly, the calculated ${D}_{0}$ is in the same order of magnitude of values reported in the literature for other organic crystalline systems^41^. We obtain a small value for the activation energy as a consequence of adopting pristine lattices. In this way, these results suggest that the value for *D*_0_ mentioned above is the limit of diffusivity in PTA lattices.

**Figure 6:**
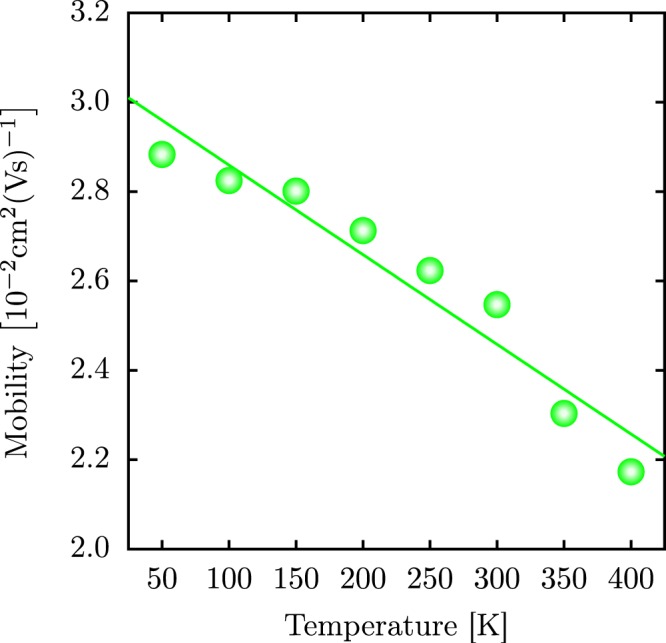
Temperature-dependent polaron mobility. The mobility is derived as an average over 1000 realizations.

Finally, Fig. 6 shows the temperature-dependent polaron mobility in 1D PTA lattices, which is derived as an average of 1000 realizations for different thermal baths. Setting *N* = 1, we can use Eqs. 3 and 4 to calculate the polaron mobilities. In this sense, the calculated mobilities have the order of magnitude of ${10}^{-2}\,{{\rm{cm}}}^{2}{({\rm{Vs}})}^{-1}$. This order of magnitude agrees with other results presented in the literature^15,16,20,42,43^. Moreover, it is worthwhile to stress that intrinsic charge mobility is difficult to obtain experimentally. Nevertheless, these theoretical results can provide reference values.

## Methods

**Figure 7:**
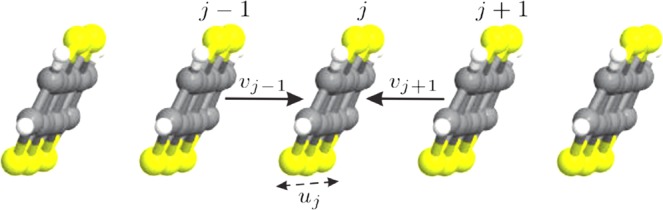
Schematic representation of a one-dimensional PTA chain, where *u*_*j*_ and *v*_*j*_ denote the intra and intermolecular degrees of freedom for a *j* site, respectively.

The approach employed here is based on a semiclassical Holstein-Peierls Hamiltonian^35^ that is used to describe the polaron dynamics in a one-dimensional PTA lattice with 100 sites and periodic boundary conditions. The model Hamiltonian considered here is a 1D version of the 2D Hamiltonian developed in ref. ^31^, where each site denotes a PTA molecule that has two degrees of freedom: an intramolecular distortion *u*_*j*_ that represents the internal deformation of a molecule in the crystal and non-local intermolecular *v*_*j*_ displacement that describes the deviation from its equilibrium position (see Fig. 7). In this sense, the model Hamiltonian used here is denoted as

6 $$H={H}_{{\rm{elec}},{\rm{intra}}}+{H}_{{\rm{elec}},{\rm{inter}}}+{H}_{{\rm{latt}},{\rm{intra}}}+{H}_{{\rm{latt}},{\rm{inter}}}$$

where

7 $${H}_{{\rm{elec}},{\rm{intra}}}=\sum _{j}\,{\alpha }_{1}{u}_{j}{\hat{c}}_{j}^{\dagger }{\hat{c}}_{j}$$

and

8 $${H}_{{\rm{elec}},{\rm{inter}}}=\sum _{j}({J}_{j,j+1}{\hat{c}}_{j+1}^{\dagger }{\hat{c}}_{j}+{\rm{H}}{\rm{.C}}.),$$

with

9 $${J}_{j,j+1}={J}_{0}-{\alpha }_{2}({v}_{j+1}-{v}_{j})\mathrm{}.$$

*J*_0_ represents the transfer integral for the pristine lattice, *α*_1_ and *α*_2_ denote the intra and intermolecular electron-phonon coupling strengths, and ${\hat{c}}_{j}^{\dagger }$ (${\hat{c}}_{j}$) creates (annihilates) a charge carrier at the *j*-site.

We use two harmonic oscillators, one for the intra and another one for the intermolecular vibrational modes, to address the lattice degrees of freedom as follows:

10 $${H}_{{\rm{latt}},{\rm{intra}}}=\frac{{K}_{1}}{2}\sum _{j}\,{({u}_{j})}^{2}+\frac{{M}_{1}}{2}\sum _{j}\,{({\dot{u}}_{j})}^{2}$$

and

11 $${H}_{{\rm{latt}},{\rm{inter}}}=\frac{{K}_{2}}{2}\sum _{j}{({v}_{j+1}-{v}_{j})}^{2}+\frac{{M}_{2}}{2}\sum _{j}{({\dot{v}}_{j})}^{2},$$

where *K*_1_ (*K*_2_) is the force constant and *M*_1_ (*M*_2_) is the harmonic oscillator mass for the intramolecular (intermolecular) degree of freedom.

The electronic dynamics is described by using the time-dependent Schrödinger equation

12 $$i\hslash {\psi }_{j}(t)=\sum _{j{\prime} }\,{H}_{j,j{\prime} }(t){\psi }_{j{\prime} }(t\mathrm{)}.$$

${H}_{j,j{\prime} }$ are the Hamiltonian matrix elements and ${\psi }_{j}(t)$ represents the electron wave function of the *j*-site at an instant *t*.

The lattice motion, in turn, is governed by the Euler-Lagrange Equations

13 $$\frac{{\rm{d}}}{{\rm{d}}t}\left(\frac{\partial \langle L\rangle }{\partial {\dot{\xi }}_{i}}\right)-\frac{\partial \langle L\rangle }{\partial {\xi }_{i}}=0.$$

To take into account the lattice effects, it is necessary to obtain the expectation value of the Lagrangean, $\langle \psi |L|\psi \rangle$, where $|\psi \rangle$ is the Slater wave function represented in the second quantization formalism by $|\psi \rangle ={a}_{1}^{\dagger }{a}_{2}^{\dagger }\cdots {a}_{n}^{\dagger }|\rangle$. The Lagrangean is,

14 $$\begin{array}{rcl}L & = & \frac{{M}_{1}}{2}\sum _{j}{\dot{u}}_{j}^{2}+\frac{{M}_{2}}{2}\sum _{j}{\dot{v}}_{j}^{2}-\left(\frac{1}{2}{K}_{1}\sum _{j}{u}_{j}^{2}+\frac{1}{2}{K}_{2}\sum _{j}{({v}_{j+1}-{v}_{j})}^{2}\right)\\  &  & +\,\sum _{j}{\alpha }_{1}{u}_{j}{\hat{c}}_{j}^{\dagger }{\hat{c}}_{j}+\sum _{j}({J}_{j,j+1}{\hat{c}}_{j+1}^{\dagger }{\hat{c}}_{j}+{\rm{H}}{\rm{.C}}.)\end{array}$$

thus,

15 $$\begin{array}{rcl}\langle L\rangle  & = & \frac{{M}_{1}}{2}\sum _{j}{\dot{u}}_{j}^{2}+\frac{{M}_{2}}{2}\sum _{j}{\dot{v}}_{j}^{2}-\left(\frac{1}{2}{K}_{1}\sum _{j}{u}_{j}^{2}+\frac{1}{2}{K}_{2}\sum _{j}{({v}_{j+1}-{v}_{j})}^{2}\right)\\  &  & +\,\sum _{j}({\alpha }_{1}{u}_{j}+{J}_{0}-{\alpha }_{2}({v}_{j+1}-{v}_{j}))\sum _{j,j{\prime} }{\psi }_{j}(t){\psi }_{j{\prime} }^{\ast }(t),\end{array}$$

where $\xi$ are the $u,v$ coordinates for a given site. The sum is realized only for the occupied states. Note also that the last equation is responsible for the connection between the electronic and lattice parts of the system. The Ehrenfest method couples these two separate approximations: the electrons are quantumly described by its time-dependent electron density (a mean-field approximation), whereas classical (Newtonian) mechanics governs the nuclei motion. This mean-field approximation breaks the microscopic correlations between the force experienced by the nucleus due to the electrons and the momentum of the nucleus. The usage of such an approach is well justified in the case of charge transport. Regarding the treatment of the nuclei as classical particles, classical treatment assumes that the number of phonons (intra and inter molecular lattice vibrations) and their related energy ($Ep$) involved is large, i.e., $Ep$ « ${K}_{B}T$. Moreover, in the case of thermally activated transport, the classical approach is justified if the activation energy of nuclear coordinates *E*_*A*_ follows the relationship ${E}_{A}$ « ${K}_{B}T$. In this sense, the Newtonian equations are

16 $${F}_{u}\equiv {M}_{1}{\ddot{u}}_{j}(t)=-\,{K}_{1}{u}_{j}(t)-{\alpha }_{1}{\rho }_{j,j}(t),$$

and

17 $$\begin{array}{rcl}{F}_{v} & \equiv  & {M}_{2}{\ddot{v}}_{j}(t)\\  & = & -{K}_{2}(2{v}_{j}(t)-{v}_{j}+\mathrm{1(}t)-{v}_{j}-\mathrm{1(}t))\\  &  & -\,\frac{{\alpha }_{2}}{{M}_{2}}({\rho }_{j;j-1}(t)-{\rho }_{j+\mathrm{1;}j}(t)-{\rho }_{j-\mathrm{1;}j}(t)+{\rho }_{j;j+1}(t)),\end{array}$$

In the equations above, ${\rho }_{j,j{\prime} }$ is the electronic density matrix, that is defined as follows

18 $${\rho }_{j,j{\prime} }={\psi }_{j}(t){\psi }_{j{\prime} }^{\ast }(t\mathrm{)}.$$

The polaron diffusion in PTA lattices is studied by modifying the approach described in ref. ^31^, particularly Eqs. 10 and 11 of that work, to include temperature effects. In this sense, a thermal bath is considered in our approach by adding thermal random forces with zero mean value $\langle R(t)\rangle \equiv 0$ and variances

19 $$\langle {R}_{j}^{{\rm{intra}}}(t){R}_{j{\prime} }^{{\rm{intra}}}(t{\prime} )\rangle \equiv 2{k}_{B}T{M}_{1}{\lambda }_{1}{\delta }_{j,j{\prime} }\delta (t-t{\prime} )$$

and

20 $$\langle {R}_{j}^{{\rm{inter}}}(t){R}_{j{\prime} }^{{\rm{inter}}}(t{\prime} )\rangle \equiv 2{k}_{B}T{M}_{2}{\lambda }_{2}{\delta }_{j,j{\prime} }\delta (t-t{\prime} ),$$

to the equations of motion for the lattice backbone, *F*_*u*_ and *F*_*v*_, within the scope of Langevin formalism. Here, we suppress the indexes *x* and *y*, as presented in the equations of ref. ^31^, since we are considering just the horizontal direction for our model 1D PTA lattice. *λ*_1_ and *λ*_2_ are included in the equations above to keep the temperature constant after a transient period, namely thermalization. Therefore, the new equations of motion for the lattice have the following form

21 $${F}_{u}{\prime} \equiv {F}_{u}-{M}_{1}{\lambda }_{1}{\dot{u}}_{j}+{R}_{j}^{{\rm{intra}}}(t),$$

and

22 $${F}_{v}{\prime} \equiv {F}_{v}-{M}_{2}{\lambda }_{2}{\dot{v}}_{j}+{R}_{j}^{{\rm{inter}}}(t\mathrm{)}.$$

Equations 21 and 22 are stochastic differential equations (SDEs) and we use the Brünger-Brooks-Karplus (BBK) integrator to solve these SDEs^44,45^. The ground state lattice geometry is obtained using the Resilient back-PROPagation (RPROP) algorithm^46^. The lattice and electronic dynamics are solved within the scope of the Ehrenfest Molecular Dynamics approach, as explained in ref. ^31^. Importantly, the Holstein–Peierls approach used here has been successfully used to study oligoacene crystals in previous researches, showing a good track record^35–37,47–52^.

Table 1 presents the set of parameters used in the simulations performed here to study the polaron diffusion in 1D PTA lattices. These parameters were obtained from theoretical and experimental studies in the literature^15,16,20,22,53–55^. The temperature regimes range from 50 to 400 K and the oscillator masses *M*_1_ and *M*_2_ (see ref. ^31^) are the masses of one and two pentathienoacene molecules, respectively. The units adopted to express the values of these masses in Table 1 are commonly used in Su-Schriffer-Heeger (SSH) based approaches. It is worthwhile to mention that an 1D approach, when it comes to PTA lattices, is a reasonable approximation since this material is highly anisotropic and may present an electronic coupling about 173 meV in one direction and less than 2 meV for the other ones^16^.

**Table 1:** Set of parameters used in the simulations to study the temperature-dependent polaron dynamics. In the units below, “as” means attosecond.

Parameter	Value
*J*_0_	173.0 meV^16,20,53^
*α*_1_	2.0 eV/Å^31^
*α*_2_	0.5 eV/Å^31^
*K*_1_	14.0 eV/Å^2^ ^31,47^
*K*_2_	0.9 eV/Å^2^ ^31,47^
*M*_1_	3.2 × 10^10^ eV (as/Å)^2^
*M*_2_	6.4 × 10^10^ eV (as/Å)^2^
*a*	3.5 Å ^15,20^
*λ*_1_	5.0 × 10^4^ as^−1^ ^48^
*λ*_2_	5.0 × 10^4^ as^−1^ ^48^
